# Supplementary material for: Knowledge and practices surrounding malaria and LLIN use among Arab, Dazagada and Fulani pastoral nomads in Chad
Source: PLoS One. 2022 Apr 14;17(4):e0266900. doi: 10.1371/journal.pone.0266900 (PMC9009653; doi:10.1371/journal.pone.0266900)
Supplement: S1 File — (DOCX) [file pone.0266900.s001.docx]

**Title: Knowledge and practices surrounding malaria and LLIN use among Arab, Dazagada and Fulani pastoral nomads in Chad**

**Authors:** Azoukalné Moukénet^1,6*^, Sol Richardson^2,3^, Kebféné Moundiné^4^, Jean Laoukolé^5^, Ngarkodje Ngarasta^6^, Ibrahima Seck^1^

**Affiliations:**

^1^Cheikh Anta Diop University, Dakar, Senegal; ^2^Malaria Consortium, London, UK; ^3^Vanke School of Public Health, Tsinghua University, Beijing, China; ^4^StraDEC Training, Research & Innovation Department, Chad; ^5^National Malaria Control Program, Chad; ^6^University of Ndjamena, Chad

**Supplement file 1**

Table 5 Descriptive statistics of variables used in the calculation of wealth index

| Variables | Arab | Daza | Fulani | Chi2 Statistic | P-value | All |
| --- | --- | --- | --- | --- | --- | --- |
|  | **(n=105)** | **(n=84)** | **(n=89)** |  |  | **(N=278)** |
| Access to potable water source | 43 (41.0) | 9 (10.7) | 18 (20.2) | 24.4 | < 0.001 | 70 (25.2) |
| Own solar kit | 26 (24.8) | 16 (19.0) | 23 (25.8) | 1.3 | 0.524 | 65 (23.4) |
| Own a post radio | 31 (29.5) | 2 (2.4) | 24 (27.0) | 24.4 | < 0.001 | 57 (20.5) |
| Own a mobile phone | 80 (76.2) | 69 (82.1) | 50 (56.2) | 16.1 | < 0.001 | 199 (71.6) |
| Own a cart tracked by animal | 66 (62.9) | 73 (86.9) | 70 (78.7) | 15.3 | < 0.001 | 209 (75.2) |
| Own motorcycle | 42 (40.0) | 6 (7.1) | 31 (34.8) | 27.4 | < 0.001 | 79 (28.4) |
| Own at least 9 caws/camels per capita | 47 (44.8) | 15 (17.9) | 26 (29.2) | 16.0 | < 0.001 | 88 (31.7) |
| Own at least 5 sheep/goats per capita | 35 (33.3) | 23 (27.4) | 35 (39.3) | 2.8 | 0.250 | 93 (33.5) |

Table 6 Kaiser-Meyer-Olkin measure of sampling and Bartlett’s test

| KMO measure of sampling | | 0.601 |
| --- | --- | --- |
| Bartlett’s test of sphericity | Approx. Khi-square | 201.927 |
|  | P-value | < 0.001 |
