# Supplementary material for: Knowledge and practices surrounding malaria and LLIN use among Arab, Dazagada and Fulani pastoral nomads in Chad
Source: PLoS One. 2022 Apr 14;17(4):e0266900. doi: 10.1371/journal.pone.0266900 (PMC9009653; doi:10.1371/journal.pone.0266900)
Supplement: Attachment — Submitted filename: Response to Reviewers.pdf [file pone.0266900.s006.pdf]

PONE-D-21-38854: Knowledge and practices surrounding malaria and LLIN use among Arab, Dazagada and Fulani pastoral nomads in Chad

Response to Reviewers

Comments to the Author

Additional Editor Comments (if provided):

This is an interesting article that provides information about practices related with LLIN and knowledge about malaria among three pastoral Chadian nomad groups. It may inform Chadian programmes for nomads to design preventive policies for this population.
Minor comments:
1) Define abbreviations when they first appear in text

Thanks for this comment. The manuscript has been reviewed accordingly.

2) Refine sentence in Line 62 since reference #11 refers to The Gambia not to Chad

Thank you. The sentence has been refine into "Among malaria prevention strategies adopted in Chad, intermittent preventive treatment have been shown to be effective in preventing malaria among pregnant women elsewhere (11)".

3) Line 138. Specify who collected the data

Thank you for this comment. Line 138 is related to sampling and we did not specify who collected data in that section. However, in section "Data collection method" we have specified in line 155-163 "The survey questionnaire was administered in February and October 2021 by three trained data collectors fluent in the local languages and used to collect data for nomad immunization programs".

4) Line 181. Refine and justify definition of good malaria practices (both conditions: to own and to sleep?). Is it enough to consider good practice to sleep under LLIN just the night before the survey?

Thank you for raising this point. We have revised the sentence as following "Good malaria prevention practices were assessed based on the ownership and use of LLINs at night".

We agree with your comment that good malaria practices are not limited to owning and sleeping under a LLIN, and we have integrated this point as a limitation of the study in the "Strengths and limitations of the study" section, on line 384: "In addition, another limitation of this study was that its assessment of good malaria prevention practices of focused on use of LLINs, to the potential exclusion of other relevant aspects of malaria prevention."

5) Line 210. 68% respondents were male. Discuss the gender implications of the results of the survey. Would it had been different if 68% of respondents were female? (Ref # 54)

Thank you for bringing up this issue, which we agree represents a 'blind spot' for our study. We have discussed the gender aspect in lines 390 – 398 "Although malaria interventions should be sensible to gender, in this study as elsewhere, we did not found an association between gender and respectively knowledge of malaria (58) and the use of LLINs (27,59). However, the aim of this study was not to assess the sensitivity of malaria interventions to gender, thus the survey sampling was not powered to integrate the gender aspect. Therefore, further studies may explore how gender norms and roles may influence the optimal design of policies and interventions aimed at improving nomadic communities' malaria prevention practices. In the meantime, regarding the potential for women to influence positively the practice of malaria preventions (60,61), it remain important to improve nomad women' awareness of malaria and to tailor malaria prevention considering gender".

6) Lines 262-266. Correct interpretation of 6 times (0.16) and 3 times (0.37) of OR

We have changed into “one-sixth times more likely …” and “around one-third times more likely…”

7) Line 294. Refine sentence, since 77% of nomads know malaria season

Yes, but the percentage 90.64% mentioned is related to those have knowledge score above 2.5 (see Knowledge of malaria line 177). The definition of knowledge is not just knowing the correct season.

8) Line 374. Refine sentence about meaning of "root causes of malaria"

Thank you, we have modified the sentence, which now calls for the need to improve “malaria awareness of Plasmodium falciparum-carrying mosquitoes as the vector for malaria transmission” among nomadic populations.

[Note: HTML markup is below. Please do not edit.]

Reviewers' comments:

Reviewer's Responses to Questions

Comments to the Author

1. If the authors have adequately addressed your comments raised in a previous round of review and you feel that this manuscript is now acceptable for publication, you may indicate that here to bypass the “Comments to the Author” section, enter your conflict of interest statement in the “Confidential to Editor” section, and submit your "Accept" recommendation.

Reviewer #1: All comments have been addressed

---

2. Is the manuscript technically sound, and do the data support the conclusions?

The manuscript must describe a technically sound piece of scientific research with data that supports the conclusions. Experiments must have been conducted rigorously, with appropriate controls, replication, and sample sizes. The conclusions must be drawn appropriately based on the data presented.

Reviewer #1: Yes

---

3. Has the statistical analysis been performed appropriately and rigorously?

Reviewer #1: Yes

---

4. Have the authors made all data underlying the findings in their manuscript fully available?

The PLOS Data policy requires authors to make all data underlying the findings described in their

manuscript fully available without restriction, with rare exception (please refer to the Data Availability Statement in the manuscript PDF file). The data should be provided as part of the manuscript or its supporting information, or deposited to a public repository. For example, in addition to summary statistics, the data points behind means, medians and variance measures should be available. If there are restrictions on publicly sharing data—e.g. participant privacy or use of data from a third party—those must be specified.

Reviewer #1: Yes

---

5. Is the manuscript presented in an intelligible fashion and written in standard English?

PLOS ONE does not copyedit accepted manuscripts, so the language in submitted articles must be clear, correct, and unambiguous. Any typographical or grammatical errors should be corrected at revision, so please note any specific errors here.

Reviewer #1: Yes

---

6. Review Comments to the Author

Please use the space provided to explain your answers to the questions above. You may also include additional comments for the author, including concerns about dual publication, research ethics, or publication ethics. (Please upload your review as an attachment if it exceeds 20,000 characters)

Reviewer #1: (No Response)

---

7. PLOS authors have the option to publish the peer review history of their article (what does this mean?). If published, this will include your full peer review and any attached files.

If you choose "no", your identity will remain anonymous but your review may still be made public.

Do you want your identity to be public for this peer review? For information about this choice, including consent withdrawal, please see our Privacy Policy.

Reviewer #1: **Yes:** Pere Millat-Martínez
